# Comparison of Long-term Outcomes of Valve-Sparing and Transannular Patch Procedures for Correction of Tetralogy of Fallot

**DOI:** 10.1001/jamanetworkopen.2021.18141

**Published:** 2021-07-27

**Authors:** Samuel Blais, Ariane Marelli, Alain Vanasse, Nagib Dahdah, Adrian Dancea, Christian Drolet, Frederic Dallaire

**Affiliations:** 1 Department of Pediatrics, Faculty of Medicine and Health Sciences, University of Sherbrooke, Sherbrooke, Québec, Canada; 2 Centre de Recherche du Centre Hospitalier Universitaire de Sherbrooke, Sherbrooke, Québec, Canada; 3 McGill Adult Unit for Congenital Heart Disease Excellence, McGill University Health Centre, Montreal, Québec, Canada; 4 Department of Family Medicine and Emergency Medicine, Faculty of Medicine and Health Sciences, University of Sherbrooke, Sherbrooke, Québec, Canada; 5 Division of Pediatric Cardiology, Centre Hospitalier Universitaire Sainte-Justine, Montreal, Québec, Canada; 6 Division of Pediatric Cardiology, McGill University Health Centre, Montreal, Québec, Canada; 7 Division of Pediatric Cardiology, Centre Hospitalier Universitaire de Québec, Québec, Québec, Canada

## Abstract

**Importance:**

The choice of the right surgical technique for correction of tetralogy of Fallot (TOF) is contentious for patients with a moderate to severe right outflow tract obstruction. The use of a transannular patch (TAP) exposes patients to chronic pulmonary regurgitation, while valve-sparing (VS) procedures may incompletely relieve pulmonary obstruction.

**Objective:**

To compare 30-year outcomes of TOF repair after a VS procedure vs TAP.

**Design, Setting, and Participants:**

This retrospective population-based cohort study was conducted among all patients with TOF born in the province of Quebec, Canada, from 1980 to 2015 who underwent complete surgical repair. Patients who received a TAP or VS procedure were matched using a propensity score based on preoperative factors in a 1:1 ratio. Data were analyzed from March 2020 through April 2021.

**Exposures:**

The study groups were individuals who received TAP and those who received VS. The VS group was further stratified by the presence of residual pulmonary stenosis.

**Main Outcomes and Measures:**

The primary outcome was all-cause mortality, with 30-year survival evaluated using Cox proportional-hazards models. Secondary outcomes included the cumulative mean number of cardiovascular interventions, pulmonary valve replacements (PVRs), and cardiovascular hospitalizations were evaluated using marginal means/rates regressions.

**Results:**

Among 683 patients with TOF (401 patients who underwent TAP [58.7%] and 282 patients who underwent a VS procedure [41.3%]), adequate propensity score matching was achieved for 528 patients (264 patients who underwent a VS procedure and 264 patients who underwent TAP). Among this study cohort, 307 individuals (58.1%) were men. The median (interquartile range [IQR]) follow-up was 16.0 (8.1-25.4) years, for a total of 8881 patient-years, including 63 individuals (11.9%) followed up for more than 30 years. Individuals who received a VS procedure had an increased 30-year survival of 99.1% compared with 90.4% for individuals who received TAP (hazard ratio [HR], 0.09 [95% CI, 0.02-0.41]; *P* = .002). Patients who underwent TAP had an increased 30-year cumulative mean number of cardiovascular interventions compared with patients who underwent a VS procedure without residual pulmonary stenosis (2.0 interventions [95% CI, 1.5-2.7 interventions] vs 0.7 interventions [95% CI, 0.5-1.1 interventions]; mean ratio [MR], 0.36 [95% CI, 0.25-0.50]; *P* < .001) and patients who underwent a VS procedure with at least moderate residual stenosis (1.3 interventions [95% CI, 0.9-1.9 interventions]; MR, 0.65 [0.45-0.93]; *P* = .02). Results were similar for PVR, with a 30-year cumulative mean 0.3 PVRs [95% CI, 0.1-0.7 PVRs] for patients who underwent a VS procedure without residual pulmonary stenosis (MR, 0.22 [95% CI, 0.12-0.43]; *P* < .001) and 0.6 PVRs (95% CI, 0.2-1.5 PVRs) for patients with at least moderate residual stenosis (MR, 0.44 [95% CI, 0.21-0.93]; *P* = .03), compared with 1.4 PVRs (95% CI, 0.8-2.5 PVRs) for the TAP group. No statistically significant difference was found for cardiovascular hospitalizations.

**Conclusions and Relevance:**

This study found that patients who underwent a VS procedure had increased 30-year survival, fewer cardiovascular reinterventions, and fewer PVRs compared with individuals who underwent TAP, even in the presence of significant residual pulmonary stenosis. These findings suggest that it is beneficial to perform a VS procedure when possible, even in the presence of moderate residual stenosis, compared with the insertion of a TAP.

## Key Points

**Question:**

Do valve-sparing (VS) operations have better long-term outcomes than transannular patches (TAP) for correction of tetralogy of Fallot (TOF) when stratifying for residual lesions after surgical treatment?

**Findings:**

In this cohort study of 528 patients with repaired TOF in the province of Quebec, Canada, from 1980 to 2015, individuals who underwent a VS procedure had statistically significantly increased 30-year survival compared with individuals who underwent TAP. Moreover, individuals who underwent a VS procedure had statistically significantly fewer reinterventions, even in the presence of moderate residual pulmonary stenosis.

**Meaning:**

These findings suggest that it is beneficial to perform a VS procedure for correction of TOF, even in the presence of moderate residual stenosis, compared with TAP repair.

## Teaser

This cohort study compares the long-term outcomes associated with valve-sparing procedures vs transannular patches for correction of tetralogy of Fallot.

## Introduction

Currently, survival following surgical repair of tetralogy of Fallot (TOF) is excellent, with more than 95% of patients reaching adulthood.^1,2,3,4,5,6^ The ideal repair of TOF results in a complete closure of the ventricular septal defect, a functioning pulmonary valve, and no residual right ventricular outflow tract (RVOT) obstruction. The insertion of a transannular patch (TAP) effectively relieves RVOT obstruction but leads to severe pulmonary valve regurgitation.^7^ Long-standing pulmonary valve regurgitation initiates a downward spiral of chronic right ventricle volume overload and right ventricle dysfunction, associated with an increased risk of exercise intolerance, heart failure, sustained ventricular arrhythmia, and sudden death.^8,9,10,11^ Right ventricle dilatation therefore usually prompts the replacement of the native pulmonary valve at some time during follow-up, with the burden of redo operations every 10 to 15 years thereafter.^12,13^ To prevent the consequences of chronic pulmonary valve regurgitation, the surgical paradigm of correction of TOF has gradually shifted toward a more aggressive approach of preserving the pulmonary annulus at the cost of residual RVOT obstruction.^7^

The benefits of valve-sparing (VS) techniques for decreasing the risk of reintervention have been demonstrated when pulmonary annulus is well developed.^5,14^ These encouraging early results led to a broadening of the indication of VS procedures for patients with an ever-increasing degree of RVOT obstruction, at the expense of a higher proportion and severity of residual pulmonary stenosis.^15^ The risk-benefit balance of VS procedures compared with TAP remains unclear in this setting even if studies^3,16,17,18,19,20^ based on multicenter surgical registries found increased survival and decreased reintervention rates among patients who underwent a VS procedure compared with those who underwent TAP.

Nevertheless, the intricate associations among the native cardiac anatomy, the surgical approach to relieve RVOT obstruction, and the residual lesions influence the trajectory of care of patients with TOF. Appropriate bias mitigation strategies must be applied when comparing the 2 procedures because patients with TAP generally have more severe RVOT obstruction and more comorbidities. Our primary objective was to use a propensity-matched cohort to compare long-term outcomes of TAP and VS techniques for surgical correction of TOF with regard to residual lesions after surgical treatment. The secondary objective was to describe the evolution of surgical management of TOF in the last 3 decades and the distribution of residual lesions after TOF repair.

## Methods

This cohort study was approved by the ethics review board of each participating institution (ie, the Centre de Recherche du Centre Hospitalier Universitaire de Sherbrooke, Centre Hospitalier Universitaire Sainte-Justine, McGill University Health Centre, and Centre Hospitalier Universitaire de Québec) and by the appropriate governing entity regulating access to administrative data in Quebec. Given the retrospective nature of the study and deidentification of the records, the requirement for individual consent was waived by all participating institutions. This study was reported in adherence to the Strengthening the Reporting of Observational Studies in Epidemiology (STROBE) reporting guideline.

### Study Design and Population

We analyzed a subset of the Tetralogy of Fallot Research for Improvement of Valve replacement Intervention: A Bridge Across the Knowledge Gap (TRIVIA) study, a retrospective population-based cohort including all patients with TOF born from 1980 to 2015 in Quebec, Canada. The detailed rationale and design of the TRIVIA study have been described previously.^21,22^ We included all patients with TOF who had at least mild pulmonary RVOT obstruction and underwent surgical correction with a VS procedure or TAP. We excluded individuals with pulmonary atresia, TOF with absent pulmonary valve syndrome, and concomitant severe heart defects.

### Data Collection

The final cohort data comprised detailed pediatric clinical data for patients from birth to age 18 years linked with more than 35 years of health care administrative follow-up data using a unique identifier. Clinical data were manually abstracted from patients’ records using a standardized collection form and included information on native anatomy, palliative interventions, corrective surgical treatment, genetic conditions, and residual lesions. Administrative data were extracted from the Quebec Congenital Heart Disease database, which combines information from the physician’s service and claims database and the hospital discharge summary database and provides systematically recorded diagnoses and health services rendered to all patients with congenital heart disease in Quebec.^23,24^

### Outcomes

Patients were followed up from corrective surgical treatment until death or 2017. The primary outcome was all-cause mortality. The causes of death were extracted from the diagnostic codes in the discharge summaries and death certificates. Deaths were stratified as in-hospital and postdischarge deaths according to the discharge date from the index hospitalization for corrective surgical treatment. Secondary outcomes were the cumulative number of cardiovascular interventions, pulmonary valve replacements (PVRs), and unplanned cardiovascular hospitalizations. Cardiovascular interventions and PVRs were identified using physicians’ and surgeons’ billing codes. Hospitalizations were restricted to unplanned hospitalizations for adverse cardiovascular events using *International Classification of Diseases, Ninth Revision *(*ICD-9*) and *International Statistical Classification of Diseases and Related Health Problems, Tenth Revision *(*ICD-10*), thus excluding hospitalizations for elective procedures. The types of interventions and the causes of hospitalization were extracted from the administrative database and stratified by study group. The algorithms used to identify causes of death, cardiovascular interventions, and hospitalizations have been described previously.^22^

### Variables of Interest

Patients were divided into 2 study groups by type of TOF repair: TAP or VS procedure. For the VS group, outcomes were further stratified by the absence or presence of moderate to severe residual pulmonary stenosis (defined by the clinician on an echocardiogram report or measured at >35 mm Hg by Doppler). The VS groups comprised patients whose repairs were done without the use of a transannular incision: patch of the RV infundibulum, patch of the main pulmonary artery, a combination of both, or no patch.

Other baseline variables of interest included age at correction, severity of native pulmonary stenosis at first postnatal follow-up, presence of a genetic condition, history of previous palliative procedure (eg, shunt or stent), presence of an atrial septal defect (ASD), and surgical era (represented by decade of birth). The severity of pulmonary stenosis was extracted from the echocardiogram report according to the transpulmonary Doppler gradient: trivial (<20 mm Hg), mild (20-35 mm Hg), moderate (35-65 mm Hg), or severe (>65 mm Hg).

### Handling of Missing Data

There were complete data for 495 patients (72.5%), and 125 patients (18.3%) had 1 variable with missing values. However, the number of missing values for variables ranged from 8 missing for history of palliative procedures (1.2%) to 110 missing for native severity of pulmonary stenosis (16.2%). We used multiple imputation with a fully conditional specification method to produce 50 imputed data sets.^25^ We included every perioperative covariable of interest listed previously. The rationale, description, and validation of the imputation model are detailed in eMethods in the Supplement.

### Statistical Analysis

All statistical analyses were performed using SAS statistical software version 9.4 (SAS Institute). For all analyses, the level of statistical significance was *P* < .05 and hypothesis tests were 2-sided. Data were analyzed from March 2020 through April 2021. Categorical variables are presented as numbers and percentages, and continuous variables are presented as mean (SD) or median (interquartile range [IQR]), depending on the variables’ distribution. We used absolute standardized mean differences (SMD) to compare the distribution of baseline variables in the TAP and VS groups. The descriptive baseline variables and descriptive outcomes (ie, surgical treatment type, cause of deaths, and cause of hospitalization) were computed in the unmatched data set, while the effect sizes of surgical groups were evaluated in the propensity-matched imputed data sets.

#### Propensity Score Matching

To decrease the potential of bias by indication, we used a 2-condition matching strategy to match patients who underwent TAP to patients who underwent a VS procedure in a 1:1 ratio. Because RVOT obstruction is the most important predictor associated with the type of surgical treatment, we used direct matching on the severity of native pulmonary stenosis in addition to propensity score matching. The propensity score was calculated using a logistic binomial regression model representing the probability of individuals undergoing a VS procedure based on baseline preoperative characteristics. We used an optimal iterative matching algorithm based on the lowest absolute difference of propensity score between matched individuals, with a maximal caliper of 0.15.^26,27^ The propensity score model is presented in detail in eMethods in the Supplement.

The propensity-matching efficacy for balancing perioperative variables between study groups was assessed using absolute differences and statistical comparison.^28^ The following criteria were used: decrease of the absolute SMD to less than 0.10 and a decrease in the *R*^2^ of the propensity score model.^29^

#### Regression Models

We compared corrective surgical groups in the propensity-matched data set using a multivariable Cox proportional hazard model for mortality and multivariable marginal means/rates models for recurrent events for interventions, PVRs, and hospitalizations. The marginal means/rates regression, which is a semiparametric extension of Cox proportional hazard, models the cumulative mean number of events per individual through time.^30,31^

We performed the analysis in each of the 50 imputed propensity-matched data sets in parallel and then computed final estimates by pooling individual estimates. Results from the Cox multivariable model are presented as hazard ratios (HR), and results from marginal means/rates models are presented as mean ratios (MR) with associated 95% CIs. We computed estimated survival and estimated cumulative mean numbers of interventions, PVRs, and hospitalizations per patient from corrective surgical treatment to 30 years after correction. The proportional hazard assumption was verified by plotting Schoenfeld residuals against time. The potential of multicolinearity was also verified prior to analysis.

#### Mitigation of Surgical Era Bias

This study spanned several decades, which allowed us to study the outcomes of surgical treatment over a long period of time. However, because surgical groups are distributed unevenly across time and the quality of care has improved over the same period, surgical year may constitute an important confounding factor. To mitigate this possibility, we included the surgical year in the calculation of the propensity score and included a covariable representing surgical year in the final models. Because the risk associated with surgical year was nonlinear, we initially divided the study period by 5-year intervals. For the propensity score model and the final outcomes models, the risks were similar for adjacent 5-year categories. They were thus combined into 10-year categories. We also tested a natural cubic spline function to evaluate the association of surgical year with outcomes as a continuous variable as a sensitivity analysis (eMethods in the Supplement).

#### Post Hoc Sensitivity Analyses

Considering that several analytical approaches may be used to decrease the potential for bias by indication, we performed additional post hoc analyses with alternative methods. We tested 2 propensity score computation methods (with or without spline functions for surgical year) and 4 models for propensity score adjustment: propensity score matching with and without subsequent covariable adjustment, inverse-probability weighting, and the inclusion of the propensity score as a covariable. We also tested direct covariable adjustment without propensity score for 9 alternative models. Additionally, we explored the presence of a modifying effect of the surgical period on the outcomes by performing sensitivity analyses stratified for era. These alternative models are described in detail in eTable 1 in the Supplement.

## Results

Among 891 patients who underwent surgical correction of TOF in the TRIVIA database, 208 patients were excluded (186 patients had pulmonary atresia or a severe concomitant heart defect, and 22 patients received a conduit), leaving 683 eligible patients. There were 282 patients (41.3%) who underwent a VS procedure for surgical correction of TOF and 401 patients (58.7%) who received a TAP. The baseline characteristics by type of surgical correction for the unadjusted, imputed, and propensity-matched data sets are presented in the Table. The TAP group, compared with the VS group, had an increased proportion of patients with severe pulmonary stenosis before correction (104 patients [25.9%] vs 56 patients [19.9%]; SMD = 0.34), a concomitant genetic condition (66 patients [16.5%] vs 38 patients [13.5%]; SMD = 0.18), and previous palliative surgical treatment (120 patients [29.9%] vs 48 patients [17.0%]; SMD = 0.31). The presence of an ASD was also more prevalent in this group (135 patients [33.7%] vs 76 patients [27.0%]; SMD = 0.17).

**Table.** Population Baseline Characteristics by Surgical Correction Technique and Adjustment

Variable	Unadjusted raw data			After multiple imputation			After multiple imputation and propensity score matching		
	No. (%)		SMD	No. (%)		SMD	No. (%)		SMD
	TAP (n = 401)	VS procedure (n = 282)		TAP (n = 401)	VS procedure (n = 282)		TAP (n = 264)	VS procedure (n = 264)	
Pulmonary stenosis									
Mild	62 (15.5)	79 (28.0)	0.34	64 (16.1)	83 (29.3)	0.32	65 (24.5)	65 (24.5)	0.00
Moderate	160 (39.9)	112 (49.7)		218 (54.3)	133 (47.3)		133 (50.5)	133 (50.5)	
Severe	104 (25.9)	56 (19.9)		119 (29.6)	66 (23.4)		66 (25.0)	66 (25.0)	
Unknown	75 (18.7)	35 (12.4)		NA	NA		NA	NA	
Atrial septal defect									
Yes	135 (33.7)	76 (27.0)	0.17	145 (36.2)	82 (28.7)	0.16	82 (31.1)	67 (25.4)	0.09
No	251 (62.6)	199 (70.5)		256 (63.8)	200 (71.3)		182 (68.9)	197 (74.6)	
Unknown	15 (3.7)	7 (2.5)		NA	NA		NA	NA	
Palliative surgical procedure									
None	277 (69.1)	230 (81.6)	0.31	278 (69.3)	231 (82.0)	0.30	204 (77.3)	213 (80.7)	0.07
≥1	120 (29.9)	48 (17.0)		123 (30.7)	51 (18.0)		60 (22.7)	51 (19.3)	
Unknown	4 (1.0)	4 (1.4)		NA	NA		NA	NA	
Genetic condition									
Yes	66 (16.5)	38 (13.5)	0.18	68 (16.7)	40 (13.8)	0.08	42 (15.9)	32 (12.1)	0.09
No	306 (76.3)	233 (82.6)		334 (83.3)	242 (86.2)		222 (84.1)	232 (87.9)	
Unknown	29 (7.2)	11 (3.9)		NA	NA		NA	NA	
Surgical era									
1980-1989	110 (27.4)	65 (23.1)	0.35	110 (27.4)	65 (23.1)	0.35	75 (28.4)	63 (23.9)	0.06
1990-1999	148 (36.9)	70 (24.8)		148 (36.9)	70 (24.8)		60 (22.7)	67 (25.4)	
2000-2010	95 (23.7)	94 (33.3)		95 (23.7)	94 (33.3)		84 (31.8)	84 (31.8)	
2010-2015	48 (12.0)	53 (18.8)		48 (12.0)	53 (18.8)		45 (17.1)	50 (18.9)	
Age at surgical procedure, median (IQR)	0.86 (0.41-1.91)	0.96 (0.50-1.80)	0.07	0.86 (0.41-1.91)	0.96 (0.50-1.80)	0.07	0.85 (0.38-2.00)	0.97 (0.51-1.86)	0.01

Abbreviations: IQR, interquartile range; NA, not applicable; SMD, standardized mean difference; TAP, transannular patch; VS, valve-sparing.

We achieved adequate propensity matching for 528 patients (264 patients who underwent a VS procedure and 264 patients who underwent TAP), who were studied in final analyses. In the study cohort, 307 individuals (58.1%) were men. The median (IQR) follow-up was 16.0 (8.1-25.4) years, for a total of 8881 patient-years, including 63 patients (11.9%) followed up for more than 30 years. The VS group was further divided according to the presence of residual stenosis after surgical treatment; 190 patients underwent a VS procedure without significant residual pulmonary stenosis, and 74 patients had at least moderate residual pulmonary stenosis after surgical treatment. After propensity score matching, the SMDs between TAP and VS groups were significantly decreased and were lower than 0.1 for all variables (eg, the SMD for the severity of native pulmonary stenosis decreased from 0.32 to 0.00), and the *R*^2^ of the propensity score model decreased from 0.095 to 0.010.

### Evolution of Surgical Approach

**Figure 1.**
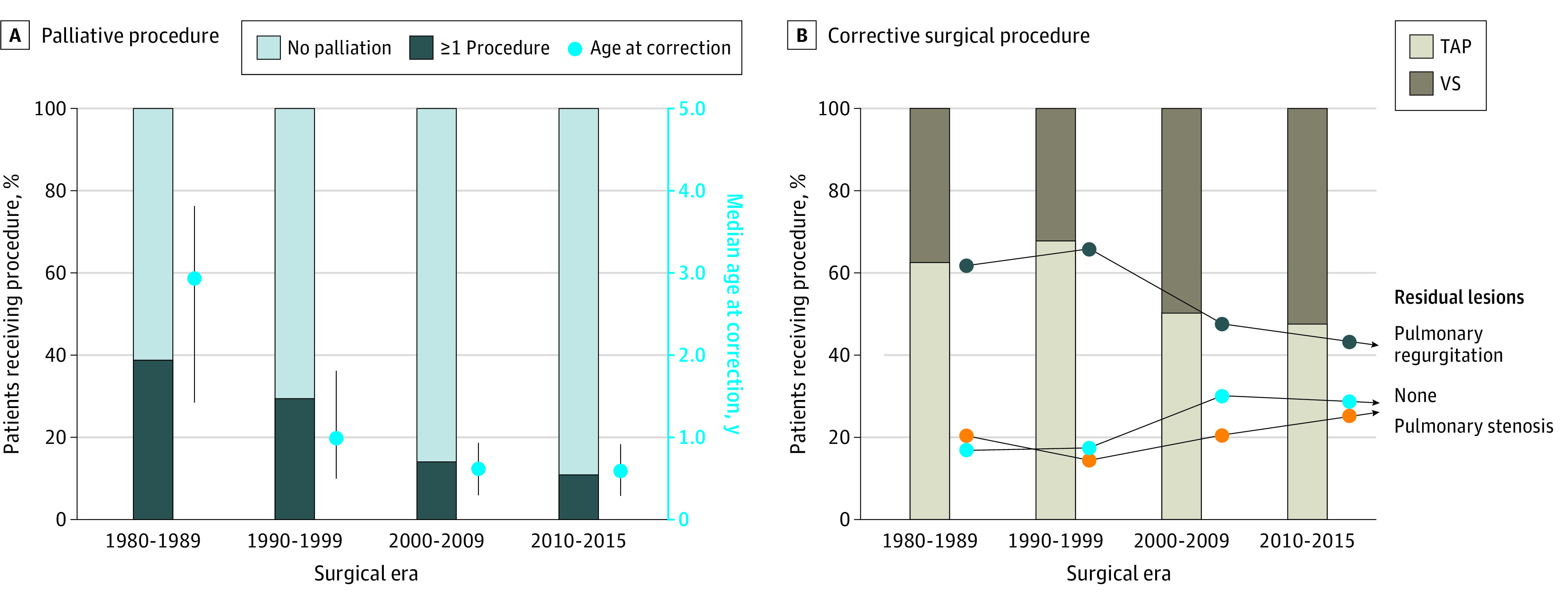
Evolution of Early Surgical Management of Tetralogy of Fallot and Residual Lesions A, The proportion of patients who underwent palliative surgical treatment and median age at correction are displayed. B, The proportion of patients who underwent transannular patch (TAP) and valve-sparing (VS) procedures and the proportion of the predominant residual lesion after corrective surgical treatment are displayed.

The evolution of surgical management and its association with residual lesions after surgical correction are presented in Figure 1. In the decades from 1980 to 2010, the proportion of patients who underwent at least 1 palliative surgical procedure decreased from 68 of 174 patients (39.1%) to 11 of 101 patients (10.9%) and the median (IQR) age at correction decreased from 2.8 (1.4-3.8) years to 0.6 (0.3-0.9) years. The proportion of patients receiving TAP decreased from 110 of 175 patients [62.9%] to 48 of 101 patients [47.5%], at the benefit of VS, which increased from 65 of 175 patients [37.1%] to 53 of 110 patients [52.5%]. During the same period, the proportion of patients with more than mild pulmonary regurgitation decreased from 97 of 156 patients [62.2%] to 45 of 101 patients [44.6%], while the proportion of patients with no significant residual lesions increased from 29 of 156 patients [18.6%] to 29 of 101 patients [28.7%]. However, the proportion of patients with more than mild residual pulmonary stenosis also increased, from 30 of 156 patients [19.2%] to 27 of 101 patients [26.7%].

### Comparison of VS Procedures vs TAP

#### Survival

**Figure 2.**
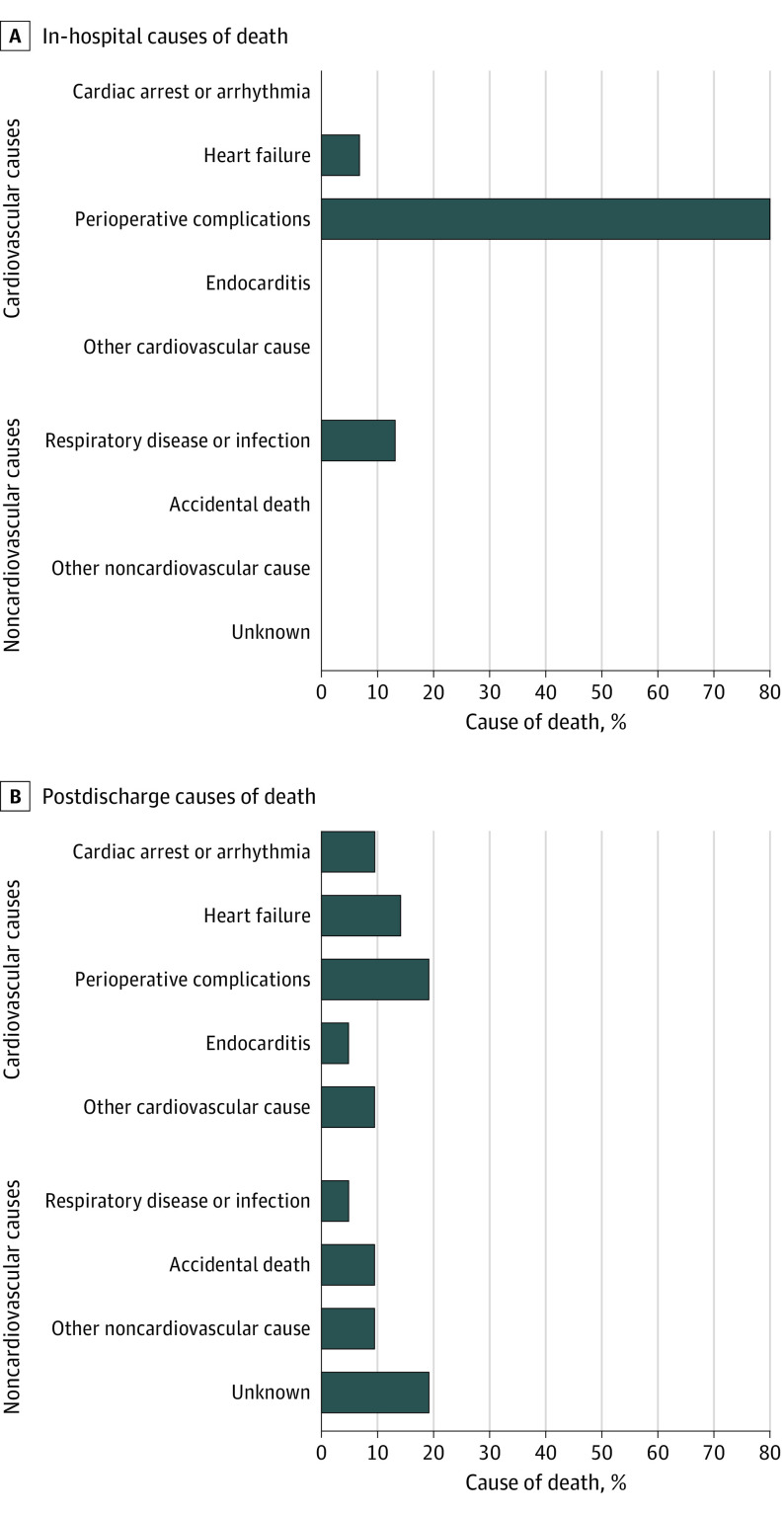
Causes of Death After Correction of Tetralogy of Fallot There were 0 patients with in-hospital death related to cardiac arrest or arrhythmia, endocarditis, other cardiovascular cause, accidental, other noncardiovascular cause, or unknown.

There were 36 deaths among 683 eligible patients (5.3%) in the unmatched cohort. The causes of death are described in Figure 2. Of them, 15 deaths (41.7%) were in-hospital deaths and 21 deaths (58.3%) occurred after discharge. Most of the in-hospital deaths were attributable to perioperative complications (12 patients [80.0%]) and respiratory infections (2 patients [13.3%]). The most common postdischarge causes of death were heart failure (3 patients [14.2%]) and perioperative complications of reinterventions (4 patients [19.1%]). There were no deaths observed in the VS group with moderate to severe pulmonary stenosis and 2 deaths in the VS group with no residual pulmonary stenosis (both owing to cardiovascular causes). Hence, these VS groups were combined to generate final estimates.

**Figure 3.**
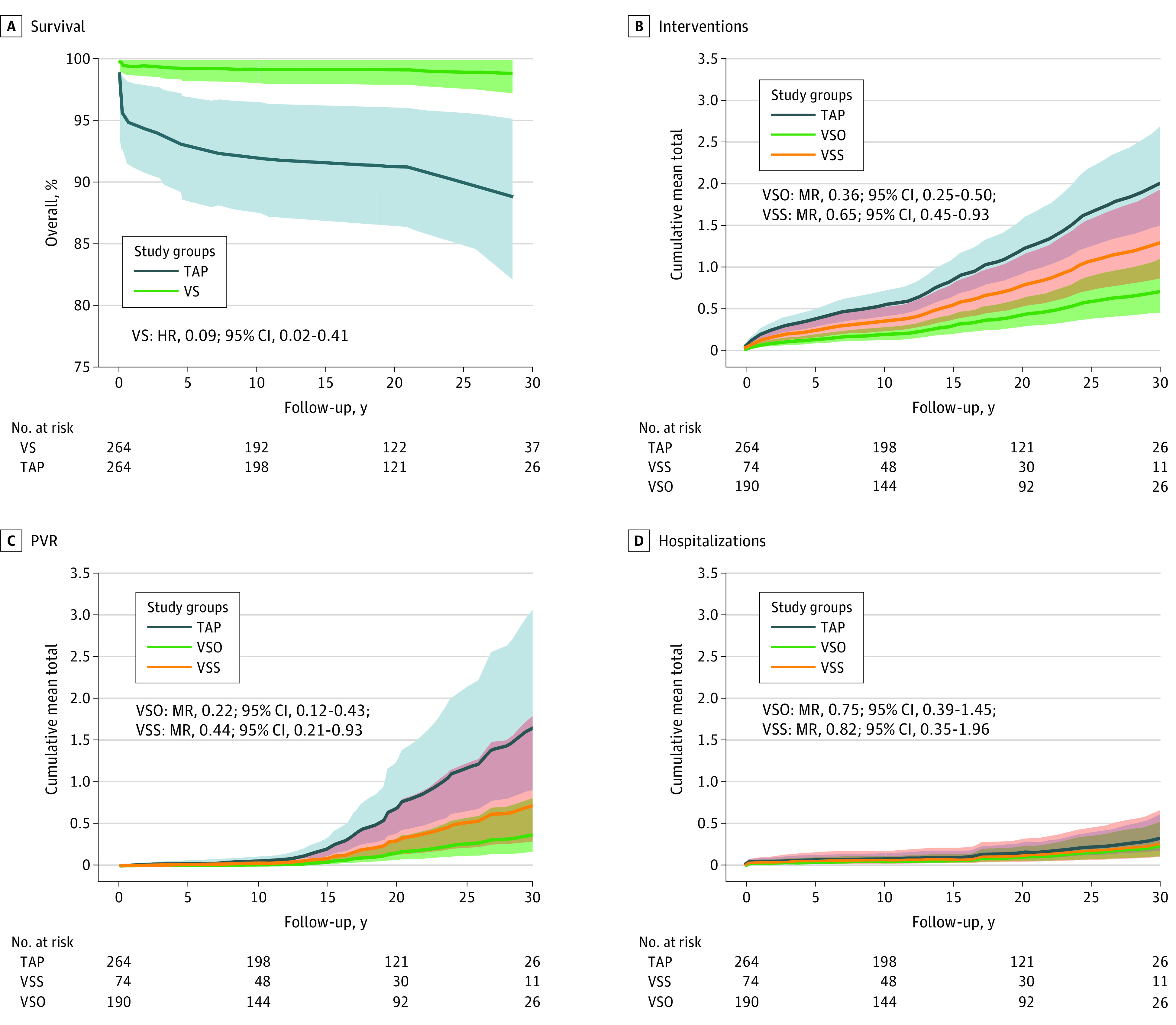
30-Year Outcomes of Surgical Correction of Tetralogy of Fallot Estimated survival and hazard ratio modeled with Cox regression are presented (A). Cumulative mean numbers and mean ratios of cardiovascular interventions (B), pulmonary valve replacements (PVR) (C), and unplanned hospitalizations for adverse cardiovascular events (D) modeled with marginal means/rates regression are presented. Transannular patch (TAP) was the reference group. HR indicates hazard ratio; MR, mean ratio; VS, valve-sparing procedure; VS0, valve-sparing procedure without significant residual pulmonary stenosis; VSS, valve-sparing procedure with more than mild residual pulmonary stenosis.

In the propensity-matched cohort, the VS group had an approximately 10-fold decreased risk of mortality compared with the TAP group (HR = 0.09 [95% CI, 0.02-0.41]; *P* = .002), translating to an increased 30-year postoperative survival of 99.1% vs 90.4%. The modeled survival rates and HRs are presented in Figure 3A, and the detailed survival estimates with 95% CIs are presented in eTable 2 in the Supplement. We observed 3 deaths among patients born after 2000. Hence, the observed difference in mortality between groups was mainly associated with earlier surgical eras. There were no deaths later than 28 years after correction so the CIs of survival could not be calculated thereafter.

#### Cardiovascular Interventions

**Figure 4.**
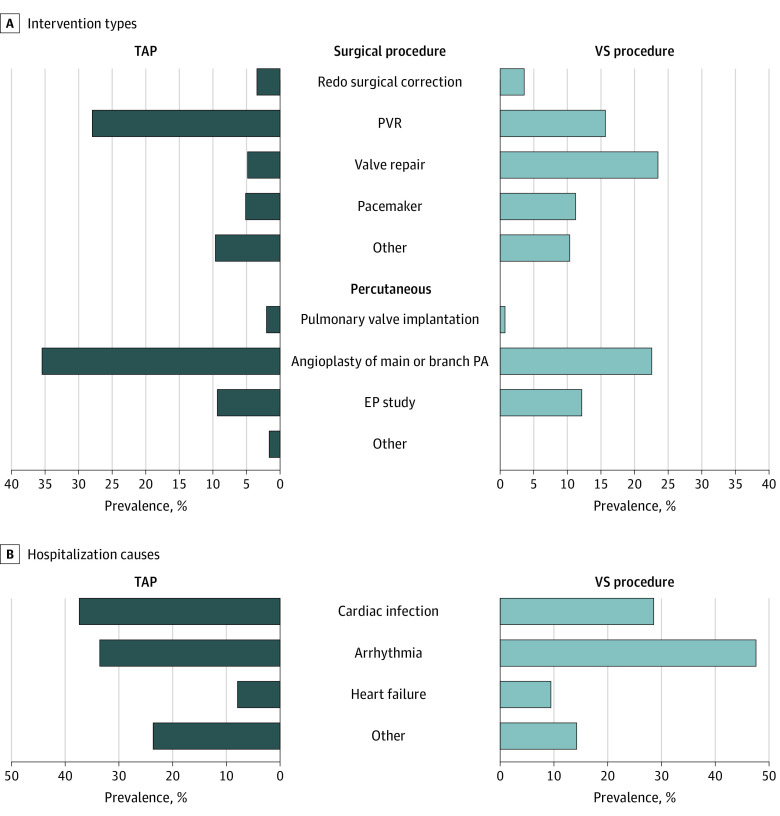
Cardiovascular Interventions and Causes of Unplanned Hospitalizations for Adverse Cardiovascular Events EP indicates electrophysiology; PA, pulmonary artery; PVR, pulmonary valve replacement; TAP, transannular patch; VS, valve-sparing.

We recorded 540 cardiovascular interventions after surgical correction in the unmatched cohort. The proportion of each type of interventions is presented in Figure 4. In both groups, the most common interventions were percutaneous angioplasty of the main pulmonary artery or branch pulmonary arteries and pulmonary valve replacements. For the TAP group, 151 of 425 interventions (35.5%) were percutaneous angioplasty of the main pulmonary artery or branch pulmonary arteries and 119 of 425 interventions (28.0%) were pulmonary valve replacements. For the VS group, 26 of 115 interventions (22.6%) were percutaneous angioplasty of the main pulmonary artery or branch pulmonary arteries and 18 of 115 interventions (15.7%) were pulmonary valve replacements.

The estimated 30-year cumulative mean numbers of interventions and MR are presented in Figure 3B, and the detailed estimates with 95% CI are presented in eTable 3 in the Supplement. Patients who underwent a VS procedure with no residual stenosis had a decreased 30-year cumulative mean number of interventions compared with patients who underwent TAP (0.7 interventions [95% CI, 0.5-1.1 interventions] vs 2.0 interventions [95% CI, 1.5-2.7 interventions]; MR = 0.36 [95% CI, 0.25-0.50]; *P* < .001). Patients with moderate to severe residual stenosis after VS correction also had a decreased mean number of interventions during the 30-year follow-up compared with patients who underwent TAP (1.3 interventions [95% CI, 0.9-1.9 interventions]; MR = 0.65 [95% CI, 0.45-0.93]; *P* = .02).

#### Pulmonary Valve Replacement

There were 147 PVRs performed during the study follow-up in the unmatched cohort. Of those, 10 PVRs (6.8%) were percutaneous valve implantations and 137 PVRs (93.2%) were performed surgically. The modeled 30-year cumulative mean numbers of PVR and MR are presented in Figure 3C, and the detailed estimates with 95% CIs are presented in eTable 4 in the Supplement. Patients who underwent a VS procedure had a decreased mean (95% CI) number of PVRs compared with patients who underwent TAP, even for those with more than mild residual pulmonary stenosis after correction. Compared with the mean of 1.4 PVRs (95% CI, 0.8-2.5 PVRs) among patients who underwent TAP, patients who underwent a VS procedure and did not have residual stenosis had a mean of 0.3 PVRs (95% CI, 0.1-0.7 PVRs; MR = 0.22 [95% CI, 0.12-13 0.43]; *P* < .001) and patients who underwent a VS procedure and had moderate to severe residual pulmonary stenosis had a mean of 0.6 PVRs (95% CI, 0.2-1.5 PVRs; MR = 0.44 [95% CI, 0.21-0.93]; *P* = .03).

#### Unplanned Hospitalizations for Adverse Cardiovascular Events

We observed 73 unplanned hospitalizations for adverse cardiovascular events during the 30-year postoperative follow-up in the unmatched data set. The causes of hospitalization are presented in Figure 4, the predicted cumulative mean numbers of hospitalizations and MRs are presented in Figure 3D, and the detailed estimates with 95% CIs are presented in eTable 5 in the Supplement. The most common causes of hospitalization were endocarditis (TAP: 19 hospitalizations [36.5%]; VS procedure: 6 hospitalizations [28.6%]) and arrhythmias (TAP: 17 hospitalizations [32.7%]; VS procedure: 10 hospitalizations [47.6%]). The 30-year cumulative mean number of hospitalizations was 0.3 hospitalizations (95% CI, 0.2-0.6 hospitalizations) for patients who underwent TAP, 0.2 hospitalizations (95% CI, 0.1-0.5 hospitalizations) for patients who underwent a VS procedure without residual stenosis (MR = 0.75 [95%CI, 0.39-1.45]; *P* = .34) and 0.3 hospitalizations (95% CI, 0.1-0.7 hospitalizations) for patients who underwent a VS procedure with at least moderate stenosis (MR = 0.82 [95% CI, 0.35-1.96]; *P* = .66). These differences were not statistically significant.

#### Post Hoc Sensitivity Analyses

The supplemental results of post hoc analyses for each outcome are presented in eTables 6 through 9 in the Supplement. The effect sizes, the 95% CIs, and the statistical significance obtained from these various approaches were all similar to the results presented previously.

## Discussion

Outcomes of TOF correction have been extensively studied, but reports of multicenter studies with long-term outcomes are scarce.^11,32,33,34,35,36,37,38^ Studies by the Pediatric Cardiac Care Consortium^3^ and Ylitalo et al^16^ reported 25-year survival of patients with TOF after surgical correction according to surgical correction techniques. A study from the European Association for Cardio-Thoracic Surgery^39^ evaluated the early risk of mortality on a large cohort of patients with repaired TOF. Interestingly, authors from these 3 large studies acknowledged that the lack of adjustment for presurgical confounding variables and postoperative RVOT status were important limitations. Here, we provided a well-adjusted comparison of TAP and VS long-term outcomes using an array of recognized statistical methods for decreasing the impact of bias by indication.

The primary strengths of our study are the large number of patients, the population-based study design, the long-term follow-up, and the array of robust statistical methods used. To our knowledge, this is the first study to include all individuals with TOF in a defined territory and time period. We believe that such unselected population-based cohorts may provide real-world evidence free of selection bias that translates into a higher generalizability of results. Furthermore, the combination of clinical and administrative data allows for obtaining detailed information on preoperative assessment, surgical techniques, and residual lesions while also benefiting from a lifelong follow-up period and systematic capture of outcomes, even for patients lost to clinical follow-up.

### Comparison of VS Procedures and TAP

#### Survival

Many studies found no difference in survival comparing TAP and VS procedures,^11,18,20,32,33,34,36,37,38,40^ which was likely in part associated with their limited sample-size or short follow-up. Recent larger studies have reported increased mortality for TAP compared with VS procedures. The 2019 Pediatric Cardiac Care Consortium study^3^ reported an overall survival of 94.5% and a 3.8-fold increased risk of mortality for the non-VS group. Similar findings were reported in the 2015 Ylitalo et al study^16^ and the 2012 European Association for Cardio-Thoracic Surgery study.^39^ We reported a comparable 30-year postoperative survival and 10-fold decrease in the risk of mortality for the VS groups compared with the TAP group, even after appropriate correction for the potential of bias by indication. However, this trend was mainly associated with earlier surgical eras.

#### Interventions and Hospitalizations

Most previous single center studies did not find a difference in the risk of reintervention comparing surgical procedures for correction of TOF.^18,20,32,38,40^ Some investigators reported that the freedom from reintervention was decreased with the use of TAP.^11,33^ Park et al^41^ reported a 2-fold decreased risk of reintervention for patients receiving a nontransannular approach compared with those receiving TAP. Smith et al^3^ found that patients with TAP had a 3.5-fold increased risk of undergoing a PVR compared with patients who did not undergo TAP. We found comparable results for the 30-year risk of cardiovascular reinterventions and specific PVR risk. Furthermore, we found that even for patients who underwent a VS procedure and had significant residual pulmonary stenosis, the cumulative number of PVRs and reinterventions were decreased compared with patients who underwent TAP, although the difference was less marked than for patients who underwent VS procedures and had no residual stenosis. It is likely that, in the long term, the number of interventions will remain lower in the VS group, given that the risk of needing redo pulmonary valve replacement in patients with TAP will increase. We also found that patients who underwent TAP often also underwent percutaneous angioplasty of the main pulmonary artery or branch pulmonary arteries, which was surprising. This could in part be associated with a higher proportion of branch pulmonary arteries hypoplasia in this group, stenosis at the supravalvar level at the junction of the TAP, or calcification and stenosis of a prosthetic pulmonary valve following PVR.

#### Clinical Implications

Our results suggest that, at the population level, a more aggressive approach to preserve the pulmonary valve annulus has been beneficial in the long term, even in the setting of an increased proportion of patients with moderate to severe residual pulmonary stenosis. There were no deaths in the subgroup of patients who underwent a VS procedure and had at least moderate residual stenosis. Some authors have suggested that a high RV to LV pressure ratio caused by residual RVOT obstruction was associated with an increased rate of reinterventions.^38,42^ A study by Gellis et al^43^ also found that all patients who underwent VS procedures and had residual stenosis eventually underwent balloon dilatation and that 30% of these patients required a conversion to a TAP. It was suggested that the optimal preoperative pulmonary annulus *Z* score cutoff above which one should perform a VS procedure was from −2 to −4.^38,41,44,45^ However, our results suggest that it is more beneficial to tolerate moderate residual stenosis in patients with TOF with suboptimal smaller pulmonary annulus undergoing VS corrective surgical treatment than to convert to a TAP surgical procedure.

### Limitations

This study has several limitations. The retrospective nature of this study increases the possibility of information bias and misclassification of study groups. To limit this, information on surgical correction was cross-validated from several sources. We did not have information on the preoperative diameter of the pulmonary valve annulus, and we assessed the native severity of pulmonary stenosis at the first outpatient visit after birth, which may have increased subsequently. This may have affected the quality of the VS procedure and TAP matching and may imply residual indication bias. We did not have consistent information on the surgical approach (ie, transatrial or transpulmonary vs transventricular), which have been shown to be associated with short-term outcomes.^39^ Because death and morbidity outcomes are evaluated separately, morbidity outcomes are subject to a competing risk bias. However, because the TAP group had the greatest risk of mortality and interventions, the effect size for morbidity outcomes would likely be greater and lead to the same conclusions should a competing risk methodology be used. Additionally, despite all efforts to mitigate indication bias, we cannot exclude the possibility that a residual bias by indication may be present.

## Conclusions

This study found a progressively more aggressive approach to preserve the pulmonary annulus in the last decades. Our results suggest that this outcome is associated with a decreased proportion of patients with severe pulmonary regurgitation and increases in the proportion of patients with no significant residual lesions and with significant residual stenosis after surgical correction of TOF. We found evidence that despite the increased proportion of patients with residual pulmonary stenosis, patients who underwent a VS procedure had increased 30-year survival and fewer cardiovascular interventions, particularly for pulmonary valve replacements. In more recent years, the difference in early survival between the 2 procedures seems to have narrowed, but the difference in the risk of reintervention persisted. Considering the limited longevity of prosthetic pulmonary valves, we hypothesize that this trend of decreased risk of interventions in patients undergoing a VS procedure will continue after the 30-year period we studied. Further research should address the optimal cutoff measure of the pulmonary annulus acceptable to perform a VS procedure while tolerating moderate residual pulmonary stenosis.

## Notes

Supplement. eMethods. **eTable 1.** Description of Post Hoc Models **eTable 2.** Estimated Survival From Corrective Surgical Treatment in Final Model **eTable 3.** Estimated Cumulative Mean Number Cardiovascular Interventions in Final Model **eTable 4.** Estimated Cumulative Mean Number Pulmonary Valve Replacements in Final Model **eTable 5.** Estimated Cumulative Mean Number Unplanned Hospitalizations for Adverse Cardiovascular Events in Final Model **eTable 6.** Mortality Hazard Ratios of Valve-Sparing Procedures vs Transannular Patch in Cox Proportional Hazard Models **eTable 7.** Mean Ratios of Cumulative Cardiovascular Interventions for Valve-Sparing vs Transannular Patch Groups in Marginal Means/Rates Models **eTable 8.** Mean Ratios of Cumulative Pulmonary Valve Replacements for Valve-Sparing Groups vs Transannular Patch Groups in Marginal Means/Rates Models **eTable 9.** Mean Ratios Cumulative No. Hospitalizations for Valve-Sparing vs Transannular Patch Groups in Marginal Means/Rates Models eReferences
