# Supplementary material for: Comparison of Long-term Outcomes of Valve-Sparing and Transannular Patch Procedures for Correction of Tetralogy of Fallot
Source: JAMA Netw Open. 2021 Jul 27;4(7):e2118141. doi: 10.1001/jamanetworkopen.2021.18141 (PMC8317016; doi:10.1001/jamanetworkopen.2021.18141)
Supplement: Supplement. — eMethods. eTable 1. Description of Post Hoc Models eTable 2. Estimated Survival From Corrective Surgical Treatment in Final Model eTable 3. Estimated Cumulative Mean Number Cardiovascular Interventions in Final Model eTable 4. Estimated Cumulative Mean Number Pulmonary Valve Replacements in Final Model eTable 5. Estimated Cumulative Mean Number Unplanned Hospitalizations for Adverse Cardiovascular Events in Final Model eTable 6. Mortality Hazard Ratios of Valve-Sparing Procedures vs Transannular Patch in Cox Proportional Hazard Models eTable 7. Mean Ratios of Cumulative Cardiovascular Interventions for Valve-Sparing vs Transannular Patch Groups in Marginal Means/Rates Models eTable 8. Mean Ratios of Cumulative Pulmonary Valve Replacements for Valve-Sparing Groups vs Transannular Patch Groups in Marginal Means/Rates Models eTable 9. Mean Ratios Cumulative No. Hospitalizations for Valve-Sparing vs Transannular Patch Groups in Marginal Means/Rates Models eReferences [file jamanetwopen-e2118141-s001.pdf]

Supplemental Online Content

Blais S, Marelli A, Vanasse A, et al. Comparison of long-term outcomes of valve-sparing and transannular patch procedures for correction of tetralogy of Fallot. *JAMA Netw Open*. 2021;4(7):e2118141.
doi:10.1001/jamanetworkopen.2021.18141

eMethods.

eTable 1. Description of Post Hoc Models

eTable 2. Estimated Survival From Corrective Surgical Treatment in Final Model

eTable 3. Estimated Cumulative Mean Number Cardiovascular Interventions in Final Model

eTable 4. Estimated Cumulative Mean Number Pulmonary Valve Replacements in Final Model

eTable 5. Estimated Cumulative Mean Number Unplanned Hospitalizations for Adverse Cardiovascular Events in Final Model

eTable 6. Mortality Hazard Ratios of Valve-Sparing Procedures vs Transannular Patch in Cox Proportional Hazard Models

eTable 7. Mean Ratios of Cumulative Cardiovascular Interventions for Valve-Sparing vs Transannular Patch Groups in Marginal Means/Rates Models

eTable 8. Mean Ratios of Cumulative Pulmonary Valve Replacements for Valve-Sparing Groups vs Transannular Patch Groups in Marginal Means/Rates Models

eTable 9. Mean Ratios Cumulative No. Hospitalizations for Valve-Sparing vs Transannular Patch Groups in Marginal Means/Rates Models

eReferences

This supplemental material has been provided by the authors to give readers additional information about their work.

© 2021 Blais S et al. *JAMA Network Open.*

eMethods

Multiple imputation model

We used the PROC MI procedure in SAS v9.4 with a fully conditional specification model for imputation of missing data. This process creates *K* datasets with plausible values for missing variables. We created *K*=50 datasets, which is cautiously higher than the generally recommended 20 to 30 datasets required to produce valid estimates in subsequent analyses [1, 2]. A higher number of datasets is now computationally feasible and likely to produce better characterization of variability introduced by the imputation process [3]. We chose the fully conditional specification approach because it does not rely on assumptions of multivariate normality, since most of the variables included in the model were categorical or binomial [4]. It uses an iterative approach by estimating a variable-by-variable conditional distribution with observed cases [5]. The model then randomly imputes a plausible value in each of the *K* datasets according to the association with other available variables. The fully conditional specification method can be used with monotone missing patterns, or with arbitrary missing data patterns as in our case [6].

We included all variables likely to be used in subsequent analyses, patients' characteristics associated with the missing variable observed values and variables associated with the probability of the variable to be missing in the model [6, 7]. We included information on the type of corrective surgery, the age at correction, the preoperative and postoperative severity of pulmonary stenosis, the postoperative severity of pulmonary regurgitation, a history of previous palliative procedure, the surgical era and the presence of a genetic syndrome in the model because they were the only variables respecting these criteria. Finally, to validate the imputation process, we visually compared the distribution of the genetic condition categories between the imputed and original datasets.

Validation of Missing Completely at Random (MCAR) assumption

The use of multiple imputation relies on an assumption that variables are missing completely at random (MCAR) which means that no association is present between observed and missing data. Since categorical variables were imputed, we opted for a multiple correspondence analysis approach to explore the presence of pattern between missing and observed data, where missing values were considered as a new category for the analysis. No relevant association were found between observed and missing data, which suggest that the MCAR assumption was respected.

Propensity score model

We compared two propensity score computation methods: the first one is the standard binomial model including surgical era as a categorical variable and the second tested a spline function with year of surgery as a continuous variable.

In both cases, the propensity score represents the probability of undergoing a valve-sparing (VS) procedure based on perioperative variables. We used a binomial regression model. The "outcome" was to undergo: (1) valve-sparing procedure or (0) transannular patch (TAP). The variables were included in the model using an ascending hierarchical approach, i.e., they were included one-by-one in the final multivariable propensity score model in a decremental order according to their effect size in a univariable analysis. Variables were kept in the model if there was a clinically or statistically significant association with the probability of undergoing a VS correction compared to TAP. The results from the standard binomial model are presented as odds ratios (OR) with their 95% confidence intervals (CI) in supplemental Table S1, below.

The second propensity score method used a cubic spline function to characterize the influence of surgical era. For this method, the surgical year was included as a continuous variable. Cubic spline functions are used to find inflexion points (knots) in the non-linear risk attributable to an independent variable without subjectively assuming categories. We tested multiple approaches: (1) knots manually placed at 5 years intervals, (2) Knots manually placed at 10 years intervals and (3) default function automatically attributing three equally-spaced knots to define inflexion points. The latter was used for the spline model because of lower post-matching R^2 and balancing of preoperative characteristics between groups. Three knots were placed at 1989, 1998 and 2007. The results from the binomial model including the spline function are presented as odds ratios (OR) with their 95% confidence intervals (CI) in supplemental Table S2, below.

Results From Final Propensity Score Model

**Variable**	**Odds ratio (OR)**	**95% CI**	**p-value**
**Severity of preoperative pulmonary stenosis**			
Mild or trivial	(Ref.)	(Ref.)	(Ref.)
Moderate	0.85	0.67 – 1.09	0.189
Severe	0.73	0.56 – 0.96	0.024
**Atrial septal defect***	0.85	0.72 – 1.02	0.075
**Genetic condition***	0.87	0.70 – 1.09	0.236
**History of palliative procedure***	0.67	0.54 – 0.83	<0.001
**Age at correction (in years)**	1.21	1.03 – 1.42	0.022
**Surgical era**			
1980–1989	(Ref.)	(Ref.)	(Ref.)
1990–1999	0.72	0.55 – 0.94	0.017
2000–2009	1.36	1.03 – 1.82	0.032
2010–2015	1.38	0.98 – 1.95	0.069

© 2021 Blais S et al. *JAMA Network Open.*

Results From Propensity Score Model Including Spline Function

Variable	Odds ratio (OR)	95% CI	p-value
**Severity of preoperative pulmonary stenosis**			
Mild or trivial	(Ref.)	(Ref.)	(Ref.)
Moderate	0.85	0.68 – 1.08	0.190
Severe	0.74	0.57 – 0.97	0.028
**Atrial septal defect***	0.84	0.71 – 1.00	0.054
**Genetic condition***	0.89	0.71 – 1.12	0.319
**History of palliative procedure***	0.69	0.55 – 0.86	<0.001
**Age at correction (in years)**	1.13	0.97 – 1.31	0.122
**Year of correction (continuous variable with spline function)±**			
Spline 1	0.9610	0.9130 – 1.0116	0.129
Spline 2 (cubic)	1.0043	1.0011 – 1.0074	0.007

*The odds ratio represents the presence compared to the absence of the condition.
±The inflexion knots were placed at years 1989, 1998 and 2007.
Legend: CI=confidence interval

Mitigation of the surgical era bias

In this study, surgical groups were not evenly distributed across eras. Subjects born after the year 2000 had a 1.4-fold increased risk of undergoing VS rather than TAP compared to the 1980-1990 decades (supplemental Table S1). Concurrently, outcomes have improved significantly when comparing recent eras to earlier eras, which is likely partly attributable to improvements in surgical techniques, cardiopulmonary bypass and cardiovascular critical care. This leads to a potential bias when comparing surgical groups. The era bias is not thoroughly described in the current literature, probably because long-term multi-decade observational studies are scarce [8, 9], but we suggest treating it as a confounding variable in analyses.

The surgical era was included in the computation of the propensity score and was also adjusted in all models by including a covariable based on the subjects' year of birth. In preliminary analyses, we used a categorical variable because we highly suspected a non-linearity of the risk attributable to surgical era. We initially divided surgical era by 5-year intervals but ended up joining adjacent categories because risks were similar both for propensity score and for outcomes. Surgical era is thus expressed as decades of year of birth.

We also tested spline functions to include year of surgery as a continuous variable to correct for era, as described in the "propensity score" section. However, it did not change final results and provided worse adjustment of preoperative baseline characteristics after propensity score matching compared to the use of categories.

In the final analyses, we used only two eras (1980–1999 and 2000–2015) because their effect size for every outcome was comparable to those obtained with the four-category variable. The effect size, hazard ratios and mean ratios of surgical era for survival and interventions final models are presented in supplemental Table S3. Subjects born after 2000 had a 3-fold lower mortality risk

© 2021 Blais S et al. *JAMA Network Open.*

compared to subjects born between 1980 and 1999, and a 1.8-fold higher risk of cardiovascular re-intervention, suggesting that re-operations are more frequently performed in recent surgical eras.

Association of Surgical Era With Outcomes

**Survival**	**Hazard ratio (HR)**	**95% CI**	**p-value**
1980–1999	(Ref.)	(Ref.)	(Ref.)
2000–2015	0.31	0.08 – 1.12	0.068
**Interventions**	**Mean ratio (MR)**	**95% CI**	**p-value**
1980–1999	(Ref.)	(Ref.)	(Ref.)
2000–2015	1.77	1.32 – 2.37	<0.001

Furthermore, stratified models for surgical era were used to explore the potential of a modifying effect of era on the associations between surgical groups and outcomes. Results from these sensitivity analyses are presented in supplemental Tables S4 and S5.

In the stratified survival analysis, there was significant heterogeneity in the effect of surgical groups between eras. Patients undergoing VS had a lower risk of mortality, independently of surgical era, but that trend was less marked and not statistically significant for subjects born after the year 2000 (supplemental Table S4). There were only 3 deaths in subjects born after the year 2000 which prevents us from concluding that there was a significant difference in survival between VS and TAP in the more recent decades. We can conclude that the observed difference in mortality between the VS and TAP groups is mainly attributable to their occurrence during earlier surgical eras (1980–1999).

In the stratified intervention analysis, we did not detect significant heterogeneity in the effect on surgical groups between eras (supplemental Table S5). Mean ratios were similar and led to the same conclusions when compared to the final model presented in the manuscript.

Risk of Mortality by Surgical Group in Stratified Model for Surgical Era

**Surgical groups**	**Hazard ratio (95% CI)**	
	**1980–1989**	**2000–2015**
TAP	(Ref.)	(Ref.)
VS	0.08 (0.01 – 0.59)	0.44 (0.04 – 5.03)

© 2021 Blais S et al. *JAMA Network Open.*

Risk of Cardiovascular Reinterventions in Stratified Model for Surgical Era

Surgical groups	Mean ratio (95% CI)	
	1980–1989	2000–2015
TAP	(Ref.)	(Ref.)
VS with no residual stenosis	0.41 (0.26 – 0.68)	0.25 (0.13 – 0.45)
VS with moderate to severe residual stenosis	0.78 (0.49 – 1.25)	0.45 (0.24 – 0.85)

Post-hoc sensitivity analyses: comparison of valve-sparing and transannular patch techniques

As described in the manuscript, patients in the transannular patch (TAP) groups proportionally have a more severe cardiovascular condition than patients in the valve-sparing (VS) groups which could bias conclusions when comparing study groups. The use of propensity scores to adjust analyses to reduce the potential of bias by indication was popularized by Austin, Rosenbaum and Rubin, but the best approach is still being debated [9-149-14]. Some authors have suggested that direct adjustment by confounding variables produced similar results compared with the use of propensity score adjustment [13, 15, 16]. It has been suggested that reproducing analyses using various methods and interpreting the results from each method would lead to more robust conclusions [17, 18].

As explained earlier, we compared two approaches to compute propensity scores: surgical era treated as a categorical variable or as a continuous variable with spline functions. We also chose to use four different post-hoc adjustment strategies to compare study groups using propensity score adjustment methods: propensity score matching with and without subsequent covariable adjustment, inverse-probability weighting and the inclusion of the propensity score as a covariable. This results in 8 different models (including the final model) depending on the combination of adjustment strategy and propensity score method. We also tested direct covariable adjustment without the use of propensity score as mentioned above. The description of each model is available in Table S6. The results from each model are presented in Tables S11 to S14.

eTable 1. Description of Post Hoc Models

	Dataset	Propensity score method	Covariable adjustment*	Description
**Model 1**	Imputed unmatched dataset	N/A	Yes	Direct adjustment of the confounding variables
**Model 2**	Imputed unmatched dataset	Standard	No	Propensity scores included as a covariable in the model
**Model 3**	Imputed unmatched dataset	Standard	No	Inverse probability weighting with propensity scores
**Model 4**	Imputed Propensity scores - matched dataset	Standard	Yes	Same as final model with additional adjustment of confounding variables in the model
**Model 5**	Imputed unmatched dataset	N/A	Yes	Direct adjustment of the confounding variables with year as spline
**Model 6**	Imputed unmatched dataset	With spline function	No	Propensity scores with year as spline included as a covariable in the model
**Model 7**	Imputed unmatched dataset	With spline function	No	Inverse probability weighting with propensity scores with year as spline
**Model 8**	Imputed Propensity scores - matched dataset	With spline function	No	PS-matched (year as spline) dataset without additional covariable adjustment
**Model 9**	Imputed Propensity scores - matched dataset	With spline function	Yes	Same as model 8 with additional adjustment of confounding variables in the model
**FINAL MODEL**	Imputed Propensity scores - matched dataset	Standard	No	PS matched dataset without additional covariable adjustment

* The confounding covariables included in the models are: The age at surgical correction, the severity of preoperative pulmonary stenosis, the presence of a concomitant genetic condition, the presence of an auricular septal defect, a history of previous palliative procedure and surgical era (or year of correction defined by a cubic spline function).

SUPPLEMENTAL RESULTS

Detailed estimates of outcomes according to the final model

We present the detailed computed estimates for each surgical group and outcome from the final model presented in the article. Results are visually represented in Figure 3 of the manuscript.

eTable 2. Estimated Survival From Corrective Surgical Treatment in Final Model

Study group	Estimated survival proportion (95% CI)			
	**5 years**	**10 years**	**20 years**	**30 years**
**TAP**	93.9% (90.4 – 97.5)	93.3% (89.4 – 97.1)	92.6% (88.4 – 96.7)	90.4% (n/a)
**VS**	99.4% (98.5 – 100)	99.3% (98.4 – 100)	99.3% (98.3 – 100)	99.1% (n/a)

© 2021 Blais S et al. *JAMA Network Open.*

eTable 3. Estimated Cumulative Mean Number Cardiovascular Interventions in Final Model

Study group	Estimated cumulative mean number (95% CI)			
	5 years	10 years	20 years	30 years
**TAP**	0.38 (0.28 – 0.50)	0.55 (0.42 – 0.73)	1.20 (0.92 – 1.56)	2.03 (1.52 – 2.71)
**VS0**	0.13 (0.09 – 0.20)	0.20 (0.14 – 0.29)	0.42 (0.29 – 0.62)	0.72 (0.47 – 1.09)
**VSS**	0.24 (0.16 – 0.37)	0.36 (0.24 – 0.53)	0.77 (0.52 – 1.13)	1.30 (0.88 – 1.93)

© 2021 Blais S et al. *JAMA Network Open.*

eTable 4. Estimated Cumulative Mean Number Pulmonary Valve Replacements in Final Model

Study group	Estimated cumulative mean number (95% CI)			
	5 years	10 years	20 years	30 years
**TAP**	0.02 (0.00 – 0.07)	0.04 (0.02 – 0.10)	0.57 (0.32 – 1.03)	1.36 (0.75 – 2.51)
**VS0**	0.00 (0.00 – 0.02)	0.01 (0.00 – 0.03)	0.13 (0.06 – 0.27)	0.30 (0.14 – 0.67)
**VSS**	0.01 (0.00 – 0.03)	0.02 (0.01 – 0.05)	0.25 (0.11 – 0.59)	0.60 (0.24 – 1.47)

© 2021 Blais S et al. *JAMA Network Open.*

eTable 5. Estimated Cumulative Mean Number Unplanned Hospitalizations for Adverse Cardiovascular Events in Final Model

Study group	Estimated cumulative mean number (95% CI)			
	**5 years**	**10 years**	**20 years**	**30 years**
**TAP**	0.06<br>(0.03 – 0.13)	0.08<br>(0.04 – 0.15)	0.16<br>(0.08 – 0.28)	0.33<br>(0.18 – 0.61)
**VS0**	0.05<br>(0.02 – 0.10)	0.06<br>(0.03 – 0.11)	0.11<br>(0.05 – 0.23)	0.24<br>(0.11 – 0.52)
**VSS**	0.05<br>(0.02 – 0.017)	0.06<br>(0.02 – 0.18)	0.12<br>(0.05 – 0.32)	0.26<br>(0.10 – 0.66)

Post-hoc sensitivity analyses: comparison of valve-sparing and transannular patch techniques

The choice of the propensity score method was based on the method that would yield the best balancing of preoperative baseline characteristics between the two surgical groups (TAP and VS). The use of spline functions was inferior to categorizing surgical era into decades for balancing era in the matched cohort (higher SMD and post-matching R^2). However, the impact on final results is marginal and compares to results presented in the article. Overall, each method yielded similar results which increases the robustness of our conclusions.

The effect sizes of VS groups compared to TAP with their associated 95% confidence interval and p-values from the post-hoc analyses are presented in Tables S11 to S14 for survival, interventions, pulmonary valve replacements (PVR) and unplanned hospitalizations, respectively. We also reported the percentage of variation between the post-hoc models effect size and the final models' effect size (e.g., Variation % = [Model 1 HR – Final model HR]/Final model HR).

© 2021 Blais S et al. *JAMA Network Open.*

eTable 6. Mortality Hazard Ratios of Valve-Sparing Procedures vs Transannular Patch in Cox Proportional Hazard Models

	HR	95% CI	p-value	Percentage of variation from final model HR
**Model 1**	0.100	(0.024 – 0.419)	0.002	6.4%
**Model 2**	0.103	(0.024 – 0.431)	0.002	9.6%
**Model 3**	0.117	(0.030 – 0.461)	0.002	24.5%
**Model 4**	0.091	(0.021 – 0.395)	0.001	3.2%
**Model 5**	0.098	(0.023 – 0.410)	0.002	4.3%
**Model 6**	0.099	(0.024 – 0.417)	0.002	5.3%
**Model 7**	0.113	(0.028 – 0.455)	0.002	20.2%
**Model 8**	0.104	(0.024 – 0.456)	0.003	10.6%
**Model 9**	0.106	(0.024 – 0.468)	0.003	12.8%
**Final model**	0.094	(0.022 – 0.408)	0.002	-

© 2021 Blais S et al. *JAMA Network Open.*

eTable 7. Mean Ratios of Cumulative Cardiovascular Interventions for Valve-Sparing vs Transannular Patch Groups in Marginal Means/Rates Models

	Study group	MR	95% CI	p-value	Percentage of variation from final model MR
**Model 1**	VS0	0.335	(0.244 – 0.461)	<0.001	5.9%
	VSS	0.607	(0.435 – 0.846)	0.003	6.5%
**Model 2**	VS0	0.347	(0.252 – 0.478)	<0.001	2.5%
	VSS	0.619	(0.443 – 0.865)	0.005	4.6%
**Model 3**	VS0	0.357	(0.251 – 0.509)	<0.001	0.3%
	VSS	0.627	0.442 – 0.889)	0.009	3.4%
**Model 4**	VS0	0.345	(0.253 – 0.499)	<0.001	3.1%
	VSS	0.608	(0.423 – 0.875)	0.007	6.3%
**Model 5**	VS0	0.340	(0.247 – 0.468)	<0.001	4.5%
	VSS	0.618	(0.444 – 0.863)	0.005	4.8%
**Model 6**	VS0	0.354	(0.257 – 0.489)	<0.001	0.6%
	VSS	0.627	0.448 – 0.877)	0.007	3.4%
**Model 7**	VS0	0.364	(0.256 – 0.520)	<0.001	2.2%
	VSS	0.652	(0.459 – 0.928)	0.017	0.5%
**Model 8**	VS0	0.364	(0.255 – 0.520)	<0.001	2.2%
	VSS	0.656	(0.453 – 0.949)	0.025	1.1%
**Model 9**	VS0	0.366	(0.258 – 0.520)	<0.001	2.8%
	VSS	0.621	(0.427 – 0.904)	0.013	4.3%
**Final model**	VS0	0.356	(0.253 – 0.503)	<0.001	-
	VSS	0.649	(0.452 – 0.930)	0.019	-

© 2021 Blais S et al. *JAMA Network Open.*

eTable 8. Mean Ratios of Cumulative Pulmonary Valve Replacements for Valve-Sparing Groups vs Transannular Patch Groups in Marginal Means/Rates Models

	Study group	MR	95% CI	p-value	Percentage of variation from final model MR
**Model 1**	VS0	0.194	(0.103 – 0.366)	<0.001	13.4%
	VSS	0.379	(0.182 – 0.791)	0.010	13.5%
**Model 2**	VS0	0.200	(0.106 – 0.377)	<0.001	10.7%
	VSS	0.378	(0.183 – 0.781)	0.009	13.7%
**Model 3**	VS0	0.223	(0.118 – 0.421)	<0.001	0.4%
	VSS	0.387	(0.193 – 0.773)	0.007	11.6%
**Model 4**	VS0	0.218	(0.113 – 0.421)	<0.001	2.7%
	VSS	0.405	(0.186 – 0.880)	0.022	7.5%
**Model 5**	VS0	0.202	(0.107 – 0.382)	<0.001	9.8%
	VSS	0.406	(0.193 – 0.853)	0.017	7.3%
**Model 6**	VS0	0.205	(0.109 – 0.388)	<0.001	8.5%
	VSS	0.407	(0.196 – 0.849)	0.017	7.1%
**Model 7**	VS0	0.230	(0.122 – 0.436)	<0.001	2.7%
	VSS	0.394	(0.190 – 0.816)	0.012	10.0%
**Model 8**	VS0	0.224	(0.112 – 0.448)	<0.001	0.0%
	VSS	0.425	(0.187 – 0.968)	0.042	3.0%
**Model 9**	VS0	0.220	(0.111 – 0.439)	<0.001	1.8%
	VSS	0.401	(0.173 – 0.929)	0.033	8.4%
**Final model**	VS0	0.224	(0.116 – 0.433)	<0.001	-
	VSS	0.438	0.207 – 0.931)	0.032	-

© 2021 Blais S et al. *JAMA Network Open.*

eTable 9. Mean Ratios Cumulative No. Hospitalizations for Valve-Sparing vs Transannular Patch Groups in Marginal Means/Rates Models

	Study group	MR	95% CI	p-value	Percentage of variation from final model MR
**Model 1**	VS0	0.686	(0.390 – 1.208)	0.191	8.2%
	VSS	0.864	(0.415 – 1.804)	0.699	4.9%
**Model 2**	VS0	0.726	(0.412 – 1.278)	0.267	2.8%
	VSS	0.799	(0.374 – 1.707)	0.563	3.0%
**Model 3**	VS0	0.714	(0.407 – 1.253)	0.241	4.4%
	VSS	0.817	(0.392 – 1.704)	0.590	0.8%
**Model 4**	VS0	0.707	(0.367 – 1.365)	0.302	5.4%
	VSS	0.860	(0.371 – 1.990)	0.724	4.4%
**Model 5**	VS0	0.683	(0.386 – 1.210)	0.192	8.6%
	VSS	0.861	(0.405 -1.829)	0.697	4.5%
**Model 6**	VS0	0.722	(0.408 – 1.279)	0.264	3.3%
	VSS	0.794	(0.364 – 1.734)	0.563	3.6%
**Model 7**	VS0	0.702	(0.395 – 1.248)	0.228	6.0%
	VSS	0.899	(0.429 – 1.884)	0.779	9.1%
**Model 8**	VS0	0.715	(0.373 – 1.371)	0.312	4.3%
	VSS	0.693	(0.278 – 1.729)	0.432	15.9%
**Model 9**	VS0	0.687	(0.362 – 1.304)	0.251	8.0%
	VSS	0.731	(0.299 – 1.784)	0.491	11.3%
**Final model**	VS0	0.747	(0.386 – 1.448)	0.388	-
	VSS	0.824	(0.347 – 1.962)	0.662	-

© 2021 Blais S et al. *JAMA Network Open.*

eReferences

1. Rubin DB. Multiple Imputation for Nonresponse in Surveys. Hoboken, New Jersey: John Wiley & Sons; 2004.
2. Pedersen AB, Mikkelsen EM, Cronin-Fenton D, et al. Missing data and multiple imputation in clinical epidemiological research. Clin Epidemiol. 2017;9:157-166.
3. Van Buuren S. Multiple imputation of discrete and continuous data by fully conditional specification. Stat Methods Med Res. 2007;16:219-42.
4. Van Buuren S, Boshuizen HC and Knook DL. Multiple imputation of missing blood pressure covariates in survival analysis. Stat Med. 1999;18:681-94.
5. Lee KJ and Carlin JB. Multiple imputation for missing data: fully conditional specification versus multivariate normal imputation. Am J Epidemiol. 2010;171:624-32.
6. Liu Y and De A. Multiple Imputation by Fully Conditional Specification for Dealing with Missing Data in a Large Epidemiologic Study. Int J Stat Med Res. 2015;4:287-295.
7. Glynn R, Laird N and Rubin DB. Multiple Imputation in Mixture Models for Nonignorable Nonresponse With Follow-ups. Journal of the American Statistical Association. 1993;88:984-993.
8. Delgado-Rodriguez M and Llorca J. Bias. J Epidemiol Community Health. 2004;58:635-41.
9. Rothman KJ, Greenland S and Lash TL. Modern Epidemiology: Lippincott Williams & Wilkins; 2008.
10. Austin PC. An Introduction to Propensity Score Methods for Reducing the Effects of Confounding in Observational Studies. Multivariate Behav Res. 2011;46:399-424.
11. Gu XS and Rosenbaum PR. Comparison of multivariate matching methods: Structures, distances, and algorithms. Journal of Computational and Graphic. 1993;2:405-420.
12. Rosenbaum PR and Rubin DB. Constructing a control group using multivariate matched sampling methods that incorporate the propensity score. The American Statistician. 1985;39:33-38.
13. Rosenbaum PR. Model-based direct adjustment. The Journal of the American Statistician. 1987;82:197-394.
14. Joffe MM, Ten Have TR, Felman HI and Kimmel SE. Model selection, confounder control, and marginal structural models: Review and new applications. The American Statistician. 2004;58:272-279.
15. Elze MC, Gregson J, Baber U, et al. Comparison of Propensity Score Methods and Covariate Adjustment: Evaluation in 4 Cardiovascular Studies. J Am Coll Cardiol. 2017;69:345-357.
16. Braitman LE and Rosenbaum PR. Rare outcomes, common treatments: analytic strategies using propensity scores. Ann Intern Med. 2002;137:693-5.
17. Wells AR, Hamar B, Bradley C, et al. Exploring robust methods for evaluating treatment and comparison groups in chronic care management programs. Popul Health Manag. 2013;16:35-45.
18. Fullerton B, Pohlmann B, Krohn R, et al. The Comparison of Matching Methods Using Different Measures of Balance: Benefits and Risks Exemplified within a Study to Evaluate the Effects of German Disease Management Programs on Long-Term Outcomes of Patients with Type 2 Diabetes. Health Serv Res. 2016;51:1960-80.

© 2021 Blais S et al. *JAMA Network Open.*
